# Informed consent: do information pamphlets improve post-operative risk-recall in patients undergoing total thyroidectomy: prospective randomized control study

**DOI:** 10.1186/s40463-016-0127-5

**Published:** 2016-02-13

**Authors:** Hussain Alsaffar, Lindsay Wilson, Dev P. Kamdar, Faizullo Sultanov, Danny Enepekides, Kevin M. Higgins

**Affiliations:** Sunnybrook Health Sciences Centre, University of Toronto, Toronto, ON Canada

**Keywords:** Informed consent, Pamphlets, Post-operative risk-recall, Total thyroidectomy and patient education

## Abstract

**Background:**

Informed consent consists of basic five elements: voluntarism, capacity, disclosure, understanding, and ultimate decision-making. Physician disclosure, patient understanding, and information retention are all essential in the doctor-patient relationship. This is inclusive of helping patients make and manage their decisions and expectations better and also to deal with any consequences and/or complications that arise. This study investigates whether giving patients procedure-specific handouts pre-operatively as part of the established informed consent process significantly improves overall risk-recall following surgery. These handouts outline the anticipated peri-operative risks and complications associated with total thyroidectomy, as well as the corrective measures to address complications. In addition, the influence of potential confounders affecting risk-recall, such as anxiety and pre-existing memory disturbance, are also examined.

**Methods:**

Consecutive adult (≥18 years old) patients undergoing total thyroidectomy at a single academic tertiary care referral centre are included. Participants are randomly assigned into either the experimental group (with pamphlets) or the control group by a computerized randomization system (Clinstat). All participants filled out a Hospital Anxiety and Depression Scale (HADS) and they are tested by the physician for short-term memory loss using the Memory Impairment Screen (MIS) exam. All patients are evaluated at one week post-operatively. The written recall questionnaire test is also administered during this clinical encounter.

**Results:**

Forty-nine patients are included - 25 of them receive verbal consent only, while another 24 patients received both verbal consent and patient education information pamphlets. The overall average of correct answers for each group was 83 % and 80 % in the control and intervention groups, respectively, with no statistically significant differences. There are also no statistically significant differences between the two groups, in both interview duration, in time between interviews, and in recall tests. No correlation is also apparent between the pre-op HADS score and the recall questionnaire overall score.

**Conclusions:**

A pre-operative thyroid surgical information pamphlet alone might not be sufficient to enhance patient test scores and optimally educate the patient on their expected care pathway in thyroid surgery. Supplementation with alternative means of patient education perhaps using emerging technologies needs to be further investigated.

**Electronic supplementary material:**

The online version of this article (doi:10.1186/s40463-016-0127-5) contains supplementary material, which is available to authorized users.

## Background

Informed consent consists of basic five elements: voluntarism, capacity, disclosure, understanding, and ultimate decision-making [1]. Physician disclosure, patient understanding, and information retention are essential in the doctor- patient relationship. This is inclusive of helping patients make and manage their decisions and expectations better and also dealing with any consequences and/or complications that arise.

There are many factors that can affect understanding and information retention. Some examples include, the language of communication, the psychological impact of diagnosis, the intellectual characteristics of an individual, the educational status, the level of general fund of knowledge, and the preexisting conditions affecting memory impairment and social support [2].

The understanding and retention of peri-operative expectations, possible complications, and appropriate corrective action plans can possibly avert catastrophic outcomes, unnecessary emergency visits, and decrease patient anxieties.

To enhance verbal communication, other means have been used such as pamphlets; multimedia and/or web based interactive media. Studies, especially in clinical trials [3], have demonstrated that the singular act of signing consent paperwork alone does not correlate with understanding the overall comprehensive picture of the consent, nor does it reflect on the overall quality of process. Total thyroidectomy, as it is one of the most common procedures in head and neck surgical practice with a widely understood risk profile, is chosen as the test procedure.

### Objectives

It is important to investigate whether giving patients procedure-specific handouts pre-operatively as part of the established informed consent process significantly improves overall risk-recall following surgery. These handouts outline the anticipated peri-operative risks and complications associated with total thyroidectomy, as well as the corrective measures to address some of those complications. In addition, the influences of potential confounders affecting risk-recall, such as anxiety and pre-existing memory disturbance are also examined.

## Methods

Consecutive adult (≥ 18 years old) patients undergoing total thyroidectomy at a single academic tertiary care referral centre are included. Patients included in the study who undergo total thyroidectomy are based on pre-operative cytopathology that is suggestive or is stratified as a high risk for thyroid cancer with Betheseda V or VI cytopathology. This also means that patients demonstrated no cognitive impairment, patients signed pre-operative consent paperwork, and patients are English-speaking, or they are able to effectively utilize available translational services. Participants are randomly assigned into either the experimental group (with pamphlet), or the control group with a computerized randomization system.

All patients are given verbal informed consent by senior staff surgeons. This included a review of possible post-operative complications and the associated risks and the benefits for the procedure. Signs and symptoms of major complications are carefully explained to the patient and the best corrective action plans explicitly outlined. In order to ensure uniformity and consistency of the verbal consent process, the senior staff surgeons involved in the study followed a standardized script (see Additional file 1: A.1). Concurrently, patients are asked to complete the Hospital Anxiety and Depression Scale (HADS) and they are screened for short-term memory loss using the Memory Impairment Screen (MIS) by the study coordinator [4].

In the experimental group, additional pamphlets are provided at the beginning of the interview, which is discussed point-by-point after patients are instructed and give time to read the pamphlet in its entirety. The pamphlet is a pre-existing pamphlet that is used at our institution. Translational services are offered to both control and experimental interventions as necessary. (See Additional file 1: A.2)

All participants underwent total thyroidectomy. All patients are also screened for renal dysfunction and they are prepared and sent home with prophylactic calcium and Vitamin D3 oral supplements in accordance with well-established clinical practice norms. Patients are evaluated one-week post-operatively for wound inspections, biochemical follow-ups, suture removals, and voice assessments. The written recall test is administered during this clinical encounter.

The recall test is a 12 - point multiple-choice test with two principal domains. The first six questions test patients on information in relation to the described post-operative risks; the second six questions test information in relation to the management of those highlighted post-operative complications and appropriate corrective action steps. (See Additional file 1: A.3).

Patient demographics, ethnicity, native language, interview duration, time to surgery while on a waiting list, HADS score, cognitive status, post-operative complications, unplanned clinic or emergency room visits, post-operative PTH, Vitamin D and ionized calcium levels are all collected. Statistical analysis are conducted with SPSS with *p* < 0.05 considered significant for a two-tailed testing.

## Results

Fifty consecutive patients are enrolled in the study by two senior head and neck surgeons. One patient withdrew from the study because he deferred his surgery. Of the forty-nine patients, 25 received verbal consent only, while 24 received both verbal consent and patient education information pamphlets. The mean age was 48 (27–77) years and 50 (34–74) years in the control and in the intervention groups, respectively. There are no statistically significant differences in age between the two groups with *P* = 0.697. None of the study population demonstrated cognitive impairments, while two patients in the intervention group required translational services. Patient demographic details are listed in Table 1.

**Table 1:** Participants demographics, cognitive status and language

	Control group	Intervention group	*P* value
Total number	24	25	
Gender			N/A
Male	6 (25 %)	11 (44 %)	
Female	18 (75 %)	14 (56 %)	
Mean age (years)	48 (27–77)	50 (34–74)	0.697
Cognitive impairment	None	None	N/A
Require translator	None	2	N/A

### Overall score

**Fig. 1:**
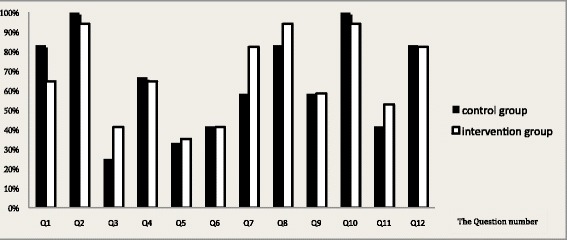
Percentages of correct answers for each question in the two groups

The overall average correct answer for each group was 83 % (25–100 %) and 80 % (35.3–94.2 %) in the control and intervention groups, respectively, with no statistically significant differences (Fig. 1). There is also no statistical significance between answers to the questions that are in the pamphlets in comparison to questions that are not in the pamphlets (Q4, Q5, Q6, Q11, and Q12).

### Overall score, interview duration and waiting time

**Fig. 2:**
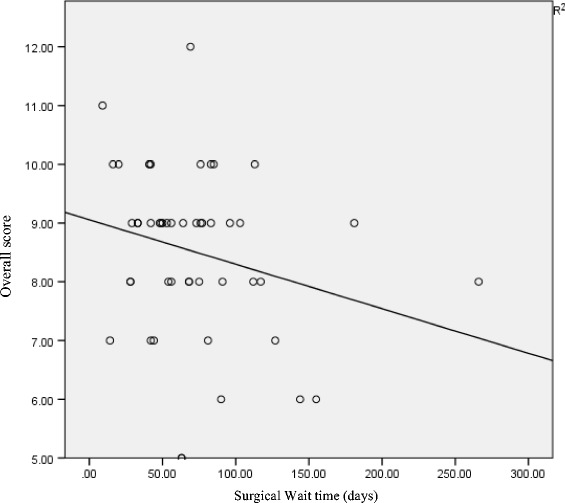
Simple linear regressions plot between surgical wait time (days) and overall scores

**Fig. 3:**
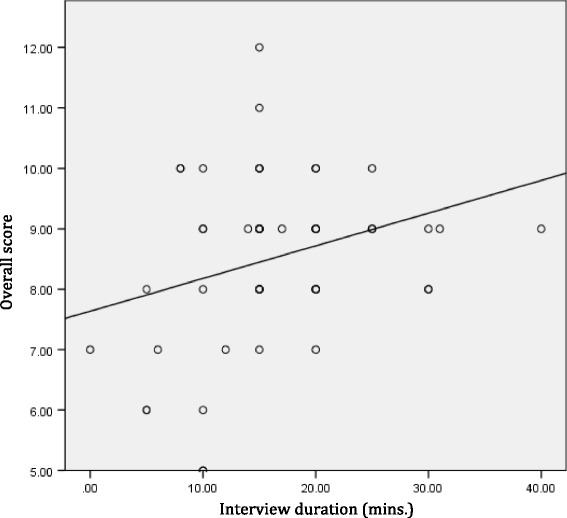
Simple linear regressions plot between duration of interview and overall score

The mean interview durations are 16.9 (5–40) and 15.8 (5–30) minutes in control and in intervention groups, respectively. The duration of two patients at the interview who required translators in the intervention group are 20 and 30 min, respectively. There are also no statistically significant differences between the two groups in regards to both interview duration and in time between interview and in recall tests (*p* = 0.663 and 0.629, respectively). This is in reference to Table 2. Further analysis between these two factors using a Pearson correlation coefficient test showed an inverse correlation between time duration with consents and with interviews in respect of an overall score correlation (−0.291) (*P* = 0.093). A notable trend towards statistical significance is also observed. For the interview duration, parametric Pearson correlation tests showed positive correlations between interview duration and overall score (0.243) with statistical significance (*P* = 0.042) (Figs. 2 and 3).

**Table 2:** Duration of interview, time lapse to test recall and post-operative complication

	Control group	Intervention group	*P* value
Mean interview duration (mins)	16.9	15.8	0.633
Duration between consent – test recall (Days)	68 (14–181)	75 (9–266)	0.629

### Relation of anxiety and depression score with overall score

All patients filled the HADS questionnaire. Pre-operative anxiety scores are borderline in 10 patients (41.6 %) and none in the control group. In the interventional group, six (24 %) are borderline and three (12 %) have abnormal scores. On the other hand, pre-operative depression scores are borderline in one patient for each group. There are no statistically significant differences between the two groups in both anxiety and in depression score with a *P* value of 0.842, 0.4163, respectively (See Table 3). There are also no correlations between the pre-op HADS score and the overall score of the recall test.

**Table 3:** Anxiety and depression scores in the two groups

	Anxiety score			Depression score		
	Control	Intervention	*P* value	Control	Intervention	*P* value
Mean score	6.54	6.36	0.842	3.5	2.92	0.416
Normal	14	16	n/a	23	24	n/a
Borderline	10	6	n/a	1	1	n/a
Abnormal	0	3	n/a	0	0	n/a

### Post-operative complication

Immediate post-operative parathyroid hormone (PTH) was within normal range (1.6–6.9 pmol/L) with no symptomatic hypocalcaemia. There are no unplanned clinic or emergency room visits during the first week post-operatively. All calcium and Vitamin D3 supplementation, that were given preoperatively as prophylaxis preoperatively, were stopped by the third post-operative based on previously established best practice protocols.

## Discussion

Aside from legal and ethical requirements, informed consent serves as an important role in patient education in regards to post-operative expectations, consequences, and complications. It is apparent that patient satisfaction correlates strongly with the amount of information received [5, 6]. The process of informed consent often occurs in one or two encounters in the outpatient clinic before surgery. This is typically done verbally and occasionally through the use of information pamphlets or through the use of audiovisual aids. This can lead to possible information overload, resulting confusion and poor information retention [7]. Previous research indicates that, just five minutes after a consultation, patients already forget half of what the doctor told them. The percentage of retained information can be raised from approximately 20 to 50% by providing visual or written information as additional support [8, 9].

This paper investigates whether presenting patients with procedure-specific handouts pre-operatively significantly improves overall risk-recall following total thyroidectomy. In addition, it examines the influence of potential confounders affecting risk-recall such as anxiety and pre-existing memory disturbances. In our study, the addition of a pre-operative handout in thyroid surgery did not improve the overall test score on post-operative recall testing between the two groups. There are no statistically significant differences between the overall test scores and the waiting times for surgery and interview durations, however there is a noticeable trend toward the negative impact of the former and positive impact of the latter. There are no correlations between pre-operative anxiety and depression and between overall recall.

Pre-operative written information demonstrates improvements in recalls on admission [10–12].

Chan et al. [10] studied the effect of written leaflets with illustrations on the ability to list three complications in 93 patients undergoing thyroid surgery. The overall recall rates are 30.6 and 50.4 % between the control and intervention groups, respectively. Similarly, in our study waiting time is not a factor between groups (average wait time to surgery of 33 days). Siau et al. reported that patients prefer to receive information leaflets and reading them subsequently demonstrated improved recall of the nature of the procedures and risks in adult tonsillectomy [13]. With regards to patient satisfaction, a cohort of patients with no significant differences between control and intervention groups for the Hospital Anxiety and Depression Scale, Clode-Baker et al. found that patients were more satisfied with information they had received and they felt less confronted by information on arrival for the hospital stay [14]. Ihedioha et al. conducted a randomized control trial to assess additional benefits of video education when combined with a written pamphlet in the psychological preparedness of patients undergoing elective colorectal surgery [15]. No differences in short-term outcomes in the hospital or in the complication rates are observed. Another important category of educational tools are web-based platforms. Advancements in social media and improved Internet access permit easy, accessible, interactive, and comprehensive tools for informed consent delivery. Farval et al. conducted a randomized control trial comparing the quality of informed consent provision in order to augment discussions with online education resources. The result is a statistically significant increase in patient knowledge with an average score of 69.25 % correct answers in the intervention group as compared to 47.38 % in the control arm with a higher patient satisfaction (*p* = 0.043). There are no statistically significant differences in anxiety scores with *P* value of 0.195.

Unlike these studies, our study has an emphasis on testing not only the inherent complication risk-recalls for medico-legal purposes, but also testing a critical educational component in respect to the corrective action plans post-operatively. Our study reveals a higher overall average recall score than other studies in both groups (80 and 83 %). This fact may relate to the multiple choice question format and study power. A limitation is that that we used a recall test which is developed for the purposes of this study, however, it has not been validated as a method of assessment.

Despite the importance of pamphlets in patient satisfaction, risk conveyance, and addressing complications, pamphlets per se might not be sufficient to optimally educate the patient on their pre-operative care pathway in thyroid surgery. Future directions point to an expansion of web-based platforms coupled with embedded informational videos which are currently being rolled out.

## Conclusion

A pre-operative thyroid surgical information pamphlet alone might not be sufficient to enhance patient test scores and optimally educate the patient on their expected care pathway in thyroid surgery. Supplementation with alternative means of patient education perhaps using emerging technologies needs to be further investigated.

## Additional file

Additional file 1:
**The script read for risk listing (See A.1).** The Pamphlet (See A.2). Risk-recall questionnaire test (See A.3). (DOCX 810 kb)

## Abbreviations

HADS: Hospital anxiety and depression scale
MIS: Memory impairment screen
PTH: Parathyroid hormone
