# Supplementary material for: Informed consent: do information pamphlets improve post-operative risk-recall in patients undergoing total thyroidectomy: prospective randomized control study
Source: J Otolaryngol Head Neck Surg. 2016 Feb 13;45:14. doi: 10.1186/s40463-016-0127-5 (PMC4752750; doi:10.1186/s40463-016-0127-5)
Supplement: Additional file 1: — The script read for risk listing (See A.1). The Pamphlet (See A.2). Risk-recall questionnaire test (See A.3). (DOCX 810 kb) [file 40463_2016_127_MOESM1_ESM.docx]

Additional file

Script V.02: October 23^rd^, 2012

THE MAJOR RISKS OF THYROIDECTOMY

- Damage to the recurrent and superior laryngeal nerves
- Damage to the parathyroid glands

DAMAGE TO THE RLN AND SLN

- Damage to the nerves occurs in <1% of thyroidectomy patients
- And can result in voice box paralysis (the inability to speak or control the pitch and volume of your voice) and difficulty swallowing and breathing

DAMAGE TO THE PARATHYROID

- The parathyroid glands are located on the back of thyroid and are responsible for regulating calcium levels in the blood
- Therefore, damage to the parathyroid glands can result in calcium deficiency called hypocalcemia
- 20% of patients will develop temporary hypocalcemia and 1% of patients will develop permanent hypocalcemia

THE SIGNS AND SYMPTOMS OF HYPOCALCEMIA

The most common indications of hypocalcemia include:

- **Paresthesia**
  - Tingling, pricking, and numbness in the hands, feet, and around the mouth
- **Tetany**
  - Twitching, stiffening, and cramping of the muscle. (This can occur anywhere in the body including the heart and throat)
- Hypocalcaemia also has been reported to cause seizures, anxieties, fatigue, and memory impairment

POST-OPERATIVE CARE INSTRUCTIONS

- Hypocalcemia is treated with calcium supplements and Vitamin D_3_
- Calcium can be taken in form of calcium carbonate (common antacid) but must be taken with food or directly after eating
- Talk to your pharmacist about which supplement is best for you and to make sure that the supplement will not affect any medication you are taking
- The risks of encountering complications following surgery can be alleviated by taking your thyroid medication regularly

**The script read for risk listing (See Additional file 1: A.1)**


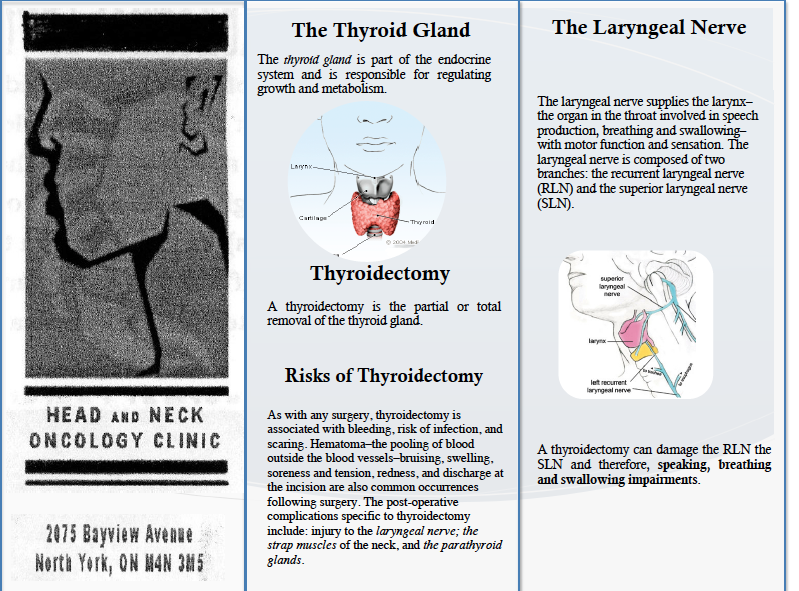

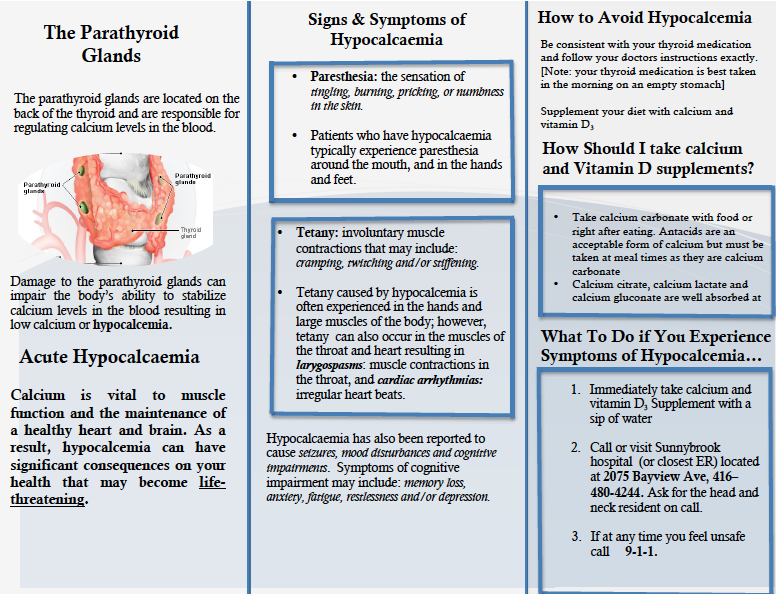


The Pamphlet (See Additional file 1: A.2)

Risk recall questionnaire test (See Additional file 1: A.3)
